# Technology Addiction: Effects of Electronic Games and Social Media Use on Academic Performance and Symptoms of Psychiatric Disorders Among School‐Age Adolescents

**DOI:** 10.1002/hsr2.71045

**Published:** 2025-07-18

**Authors:** Mosiur Rahman, Prosannajid Sarkar, Syed Emdadul Haque, G. M. Rabiul Islam, Md. Nuruzzaman Haque, Tapan Kumar Roy, Farhana Akhter Liza, Anika Tabashsum, Md. Rashed Alam, Mahmudul Hasan, Izzeldin Fadl Adam, Nguyen Huu Chau Duc, Chowdhury Mashrur Mahdee, Jannatul Fardos Asha

**Affiliations:** ^1^ Department of Population Science and Human Resource Development University of Rajshahi Rajshahi Bangladesh; ^2^ Dr. Wazed Research and Training Institute Begum Rokeya University Rangpur Bangladesh; ^3^ UChicago Research Dhaka Bangladesh; ^4^ Department of Food Engineering and Tea Technology Shahjalal University of Science and Technology, Bangladesh Sylhet; ^5^ Department of Epidemiology Faculty of Public and Environmental Health, University of Khartoum Khartoum Sudan; ^6^ Department of Pediatrics Hue University of Medicine and Pharmacy, Hue University Hue City Vietnam

**Keywords:** academic performance, adolescents, depression, electronic games, social media

## Abstract

**Background and Aims:**

There is conflicting research regarding the negative effects of electronic gaming and addictive social media use on psychological disorders and academic achievement. Therefore, we aimed to examine the association between social media use and electronic gaming addiction with depression and academic achievement.

**Methods:**

This investigation is cross‐sectional. The selection process involved 800 adolescents enrolled in grades 9 and 10 across four public and private high schools in the Rajshahi City Corporation. The outcomes of interest were depression and academic performance. Addiction to playing electronic games and using social media (mainly Facebook) were exposure of interest.

**Results:**

From the total sample, 9.3% and 28.3% reported that they were addicted to electronic games and social media use. The mean scores for depression and academic achievement were 10.2 and 3.2, respectively. For one unit increase in depression scores, the odds of addiction to playing electronic games and using social media increase by a factor of 1.14 and 1.12. Additionally, it was found that for one unit an increase in academic performance scores, the odds of addiction to playing electronic games and using social media decrease by a factor of 0.67 and 0.71.

**Conclusions:**

Adolescents who are more likely to engage in addictive behaviors such as playing electronic games or using social media should be the focus of public health initiatives, as they are more susceptible to depression along with poor academic accomplishment.

## 1 Introduction

With the expansion of high‐tech devices (e.g., smartphones, tablets, and computers) during the last two decades, the usage of modern online technologies such as electronic games and social media has grown in popularity among people of all ages, but especially among school‐going adolescents [1, 2]. Approximately 95% of adolescents between the ages of 13 and 17 worldwide report using social media [3], with over a third stating they use it “almost constantly.” According to a recent meta‐analysis spanning 33 nations, the combined prevalence of adolescent addiction to electronic gaming was 8.8% [4]. According to studies in Bangladesh 80.9% of adolescents spend more than 6 h per day on modern electronic media such as computers, the internet, and electronic games, which is more time than they spend in school or with friends [5]. Apart from online gaming, internet‐based social media platforms (e.g., Facebook) are widely used, with more than 50% of Bangladeshi adolescents utilizing these platforms many times each day [5].

Electronic games and social media use have recently been viewed as crucial not just for improved academic performance, but also for self‐expression, sociability, creativity, and amusement for children and adolescents [6, 7]. Although this technology has many great aspects, there have been concerns raised about excessive use, including the possibility of users becoming “addicted” to utilizing such technologies [8, 9]. “Being too concerned about online activities, driven by an uncontrollable need to perform the behavior, and investing so much time and effort to it that it hinders other essential life areas” is how addictive use is defined in this context [10].

Addiction to playing electronic games and using social media have been shown to have negative consequences such as decreased sleep time [11, 12], decreased physical activity [13, 14], frequent headaches [15], musculoskeletal pain [15], and functional impairments [11, 12] in children and adolescents' daily lives. Some research also looked at the relationship between excessive use of electronic games and social media and symptoms of psychiatric illnesses (such as attention deficit hyperactivity disorder, obsessive‐compulsive disorder, anxiety, and depression) [16, 17, 18, 19, 20, 21]. and adolescent academic achievement [22, 23, 24]. However, the results of those studies were inconsistent; while some revealed harmful impacts of social media and electronic gaming addiction on symptoms of psychiatric disorders [18, 19, 20, 21] and adolescent academic achievement [23], others found no link [16, 17, 24]. These conflicting results highlight the urgent need to better understand the impact of social media and electronic gaming on adolescents' development, notably academic performance, and psychiatric symptoms.

Despite many international studies and a smaller number in Bangladesh, the existing literature has several systematic limitations, particularly when it comes to the relationship between academic performance and symptoms of psychiatric disorders in school‐going adolescents who are addicted to social media and electronic games. First, most previous studies have been conducted in developed country settings [16, 17, 18, 22], which may include cultural differences such as school‐going adolescents' lifestyles, academic demands, living conditions, and school environment, which may affect academic performance, symptoms of psychiatric disorders, electronic gaming, and social media. Second, most previous research has looked at the link between electronic gaming and social media addiction and psychiatric disorders as well as academic performance in university students and the general adult population. Third, most studies in Bangladesh [19, 20] are based on the Corona Virus Disease 2019 (COVID‐19) pandemic period. Finally, differential effects of electronic games and social media use with academic achievement and psychiatric disease symptoms have only been explored and compared in a few studies [18, 22]. Electronic gaming and social media use may have different activity demands and behavioral patterns, with gaming typically requiring more rapid reactions and sustained attention over a longer period than social media use. Therefore, academic performance and psychological symptoms may be affected differently. As a result, little is known about comparing their effects, as well as the combined effects on the academic performance and symptoms of psychiatric disorders among the school‐going adolescents in question.

The objectives of this study are to assess the association between adolescent students' (1) addiction to electronic games with depression and academic performance; and (2) social media addiction with depression and academic performance.

## 2 Methods

### 2.1 Design, Settings, and Participants

This is a cross‐sectional study. Students in grades 9 through 10 between 14 and 15 years of age were our core demographic. From the Rajshahi City Corporation, a total of four high schools were chosen at random. Half of the schools were chosen from government high schools and the other half from private high schools to determine if there were any disparities between the two types of high schools. A boy's and a girl's high school were chosen from each type of high school to further determine whether there was any sex discrepancy.

**Figure 1:**
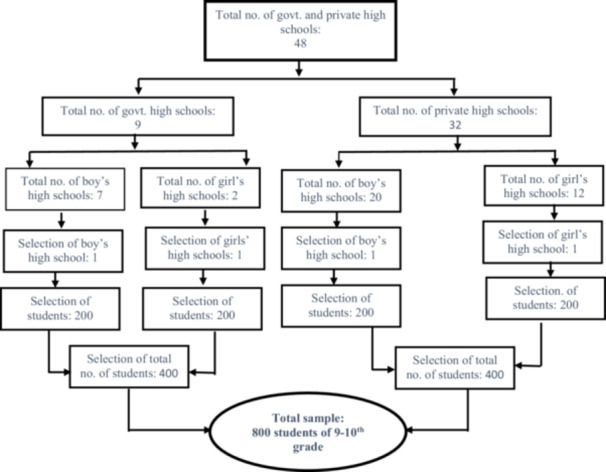
Selection of the sample.

The information was obtained using a three‐stage sampling strategy. Four public and private high schools were included in the initial phase (two government: one boy and one girl's high school; two privates: one boy and one girl's high schools were chosen). 200, 9th‐ through 10th‐graders from each school were randomly selected for the second stage (typically each grade contains 150–200 students). Eight hundred school‐aged adolescents were picked for the last round (Figure 1). The following inclusion criteria were used to choose students: (1) enrollment in a secondary high school in Rajshahi City Corporation; (2) having a Facebook account and using electronic games. Possessing a self‐reported diagnosis of neurological disease or psychological issues served as the exclusion criterion.

The sample size was estimated to be 384 using the formula: *n* = *Z* ^2^ *P*(1−*P*)/*d* ^2^, considering the confidence interval at 95% (*Z* = 1.96), population percentage at 50% (*p* = 0.5), and margin of error at 5% (*d* = 0.05%). Here, *P* stands for suggested prevalence, *Z* for statistic related to level of confidence, *n* for sample size, and *d* for precision (matching to effect size). To reduce the effects of nonresponse and to increase the study's power, the sample size was further raised to 400. We thus plan to survey a minimum sample of 800 school‐going adolescents in government and private high schools (government high schools: 400; private high schools: 400).

### 2.2 Data Collection

The data were collected during the academic session 2022–2023. A background information sheet and the questionnaires in Bangla (Bangladesh's native language) were included in the survey. The following information was collected through face‐to‐face interview with structured questionnaire: (1) sociodemographic; (2) lifestyle and online use behaviors; and (3) school level, depression, and educational performance characteristics. The questionnaires were written in English and then translated into Bangla. Experts and volunteers assessed the translation, and pilot research was done to validate the questionnaire.

### 2.3 Data Quality Assurance

Adequate design ensured data quality, and the questionnaire was pre‐tested on 10% of the overall sample (*n* = 80) who did not participate in the survey. After the questionnaires had been amended to remove any ambiguities, the final survey was carried out. Two days of intense training were provided to the data collectors to familiarize them with the tools and processes of data gathering. Every day, the principal investigator (PI) double‐checked the collected data for completeness, accuracy, and clarity. The PI also kept an eye on the data collection process. Four completely competent (out of eight) and experienced female field researchers were employed to conduct all girls' high schools because half of our sample were drawn from these institutions.

### 2.4 Measuring Variables

#### 2.4.1 Outcomes

The study's core outcomes of interest were academic achievement and signs of psychological problems. In this study, depression served as a stand‐in for a psychiatric disorder's result. The participants were screened for depression using the validated Bangla version of the patient health questionnaire (PHQ‐9) [25, 26].

The Patient Health Questionnaire has a 9‐item depression subscale called the PHQ‐9. Every PHQ‐9 depression item describes a single symptom that matches a single DSM‐IV diagnostic criterion for major depressive disorder (e.g., “having trouble concentrating,” “feeling tired or lacking energy,” and “having little interest or pleasure in doing things.” Using a 4‐point Likert scale, participants assessed how frequently they had each of the nine symptoms over the previous 2 weeks (0 = not at all, 1 = several days, 2 = more than half of the days, and 3 = virtually every day). Every item on the Likert scale contains four points. The nine items are combined, and the scores range from 0 to 27, with higher scores indicating a higher severity of depression.

Adolescent's academic performance was evaluated using an annual average score that was generated using the results of two consecutive semesters of the year's average grade point for the academic year 2021–2022.

#### 2.4.2 Exposure

Among the many online technology behaviors, we were focused on electronic gaming and social media use (specifically, Facebook) in our study.

The Bergen Social Networking Addiction Scale (BSNAS) is an adaptation of the Bergen Facebook Addiction Scale (BFAS) [27] which incorporates six items that highlight key addiction features (i.e., salience, conflict, mood alteration, withdrawal, tolerance, and relapse) was used to determine whether an individual was addicted to the use of social media. Each question was answered on a 5‐point Likert scale ranging from very seldom (1) to very often (5), generating a composite score of 6 to 30, based on past experiences (e.g., “How often have you tried to cut down on your usage of social media without success?”). The BFAS has been translated into several languages [28, 29] including Bangla [30] and has proven to have adequate psychometric characteristics in several investigations. Higher BFAS scores suggested a higher level of social media use. Our study's cut‐off score of 18 or higher suggested that social media use is problematic or addictive, in line with others research conducted in Bangladesh [31, 32]. The internal consistency of the BSMAS in the present study was 0.79.

The validated Bangla version of nine‐item Internet Gaming Disorder Scale‐Short Form (IGDS9‐SF) [33] was used to measure the addictive to electronic gaming. The items are rated using a 5‐point Likert scale from 1 (*Never*) to 5 (*Very often*). A total score was obtained by summing responses with a range from 9 to 45 points, with higher scores reflecting greater IGD severity. A cut‐off score of more than 21 was considered if electronic gaming is harmful or addictive [33].

“Do you feel the need to spend an increasing amount of time engaged gaming to get satisfaction or pleasure?” is an example question. The internal consistency of the IGDS9‐SF in the present study was 0.89.

#### 2.4.3 Confounder

Participant's age, gender, educational level, parental education, socioeconomic status (SES), family type, sleeping quality, duration of electronic games play, duration of social media use, preferred devices, physical activities, school attendance activities, school types, teacher‐student relationship were be treated as confounding variables.

### 2.5 Statistical Analyses

Descriptive statistics were used to compile a list of the participants' characteristics. Categorical variables were presented as percentage; continuous variables such as depression and academic performance were presented as mean ± standard deviation. To determine sociodemographic differences, lifestyle and online behavior, and school level characteristics in addiction to playing electronic games and using social media, we employed *χ* ^2^ analysis. To determine the significant differences between depression scores and academic performance in relation to addiction to social media and electronic game playing, a *t*‐test was used in this study. Next, multivariable logistic regression analyses were carried out to determine the relationship between our exposures (addictive to electronic game vs. none and addictive to social media use vs. none) and the likelihood of our outcome variables. All the covariates were entered simultaneously into the multiple regression models. All statistics were tested using two‐tailed with significance level set at *p*‐value < 0.05. Data of this study was reported in accordance with the Strengthening the Reporting of Observational Studies in Epidemiology guidelines. All statistical analyses were conducted using Stata version 14 (Stata Corp., College Station, TX).

### 2.6 Ethical Considerations

Ethical Committee of the Institute of Biological Sciences, University of Rajshahi provided its approval (Approval number: 336 (16)/320/IAMEBBC/IBSC). The parents' and students written informed consent was acquired. Before the survey commencement, a project information sheet explaining the study aims, survey structure, and confidentiality assurance was provided to the participants. Participants were only allowed to access the survey after indicating on a consent form that they were willing to participate. We took all mandatory steps (based on both national and worldwide recommendations) to protect staff members and participants in the event of a COVID‐19 pandemic. All study procedures were carried out in conformity with the principles of the 2013 revision of the Declaration of Helsinki.

## 3 Results

### 3.1 Descriptive Statistics

Table 1 shows descriptive statistics of sociodemographic, lifestyle and online use behaviors, school level, depression, and educational performance‐related characteristics by addiction to playing electronic games and using social media among the school‐going adolescents. A total of 800 school‐going adolescents were selected for this study. Nearly half of the respondents (48.9%) were female, 54.3% were in 9th grade of their educational level, and 55.5% were 15 years old. Regarding maternal education, about 25% of mothers have no education and 13% of fathers have no education. Most of the students (80.8%) lived in a nuclear family and approximately 52% of students' sleeping duration was normal. Most of the students' daily game play and daily social media use were < 2 h and most the preferred device for game play and social media use was having a mobile phone.

**Table 1:** Descriptive statistics of sociodemographic, lifestyle and online use behaviors, school level, depression and educational performance related characteristics by addiction to playing electronic games and using social media among the school going adolescents (*n* = 800).

Characteristics	*n* (%)	Addictive to electronic gaming		Addictive to social media	
		%	*p* value	%	*p* value
Gender					
Male	51.1	12.2	0.003	32.6	0.012
Female	48.9	6.4		24.0	
Educational level, grade					
9th	45.7	8.4	0.179	25.2	0.049
10th	54.3	10.2		31.4	
Age, yrs					
14	44.6	8.9	0.221	26.5	0.124
15	55.5	9.7		30.1	
Mothers' education^1^					
No education	25.0	13.8	< 0.001	36.1	0.032
Primary	22.0	10.3		32.3	
Secondary	22.0	8.2		27.2	
Higher	31.0	4.9		17.6	
Father's education					
No education	13.0	9.4	0.098	31.2	0.445
Primary	26.0	10.8		28.3	
Secondary	28.0	8.9		27.2	
Higher	33.0	8.1		26.5	
SES^2^					
Poor	32.1	10.3	0.512	32.5	0.685
Middle	33.3	7.7		27.8	
Rich	34.6	9.2		24.6	
Family type^3^					
Nuclear	80.8	9.9	0.392	29.9	0.878
Joint	19.2	8.7		26.7	
Sleeping duration^4^					
Normal	51.9	6.1	< 0.001	22.3	0.013
Less than normal	13.5	13.1		34.4	
More than normal	34.6	8.7		27.7	
Daily game play, h					
< 2	52.0	4.7	< 0.001	22.8	0.023
2–4	28.0	9.1		27.2	
> 4	20.0	14.1		34.9	

Characteristics	%	Addiction to playing electronic games		Addiction to using social media	
		%	*p*‐value	%	*p* value
Daily social media use, h					
< 2	47.0	5.8	< 0.001	18.7	< 0.001
2–4	35.0	9.6		24.2	
> 4	18.0	12.5		42.0	
Preferred device					
Mobile phone	77.8	11.8	0.338	29.7	0.442
Other	33.2	7.1		26.9	
Recommended physical activities^5^					
No	60.1	12.3	< 0.001	30.8	0.878
Yes	39.9	6.3		25.8	
Types of school					
Government	52.9	9.8	0.337	30.4	0.079
Private	47.1	8.8		26.2	
School attendance activities					
Almost absent	17.0	14.3	< 0.001	33.7	0.039
Sometimes	39.0	8.3		25.6	
Never absent	44.0	5.3		21.6	
Teacher‐student relationship					
Poor	20.0	13.9	< 0.001	30.8	0.567
Neutral	45.0	8.9		28.2	
Good	35.0	5.1		25.9	
Depression					
Mean (SD)	10.2 (6.7)	11.8 (7.5)	< 0.001	12.0 (6.8)	0.001
Academic performance					
Mean (SD)	3.1 (0.57)	2.8 (0.51)	< 0.001	2.7 (0.49)	< 0.001
Prevalence		9.3		28.3	

*Note:* ^1^Maternal educational level was defined in terms of the formal education system of Bangladesh: no education (0 years), primary (1–5 years), secondary (6–10 years) or completed high secondary or above (11 years or more). ^2^SES was constructed from data on 24 household assets. Each household was assigned a score for each asset using principal component analyses, and the scores were divided into tercile; each tercile was designated a rank, from one (poor) to three (rich). ^3^A nuclear family is a family that includes two married parents and their children, everyone living under one roof. There can be any number of children in a nuclear family. A joint family is a family that consists of various generations—grandparents, parents, and children. ^4^Sleeping duration: normal 6–7 h, less normal < 6 h and more than normal ≥ 8 h. ^5^Considered adherent to the recommended physical activity if [moderate physical activity + vigorous physical activity × 2] ≥ 60 min per day; (moderate e.g., conventional walking, bicycling with light effort, gardening, light exercises, e.g., cleaning house, involvement in games; vigorous physical activities e.g., running, jogging/running, bicycling with greater effort, fast swimming, team sports, e.g., football, volleyball, or basketball),

A significant proportion of the students do not follow recommended physical activities (60.1%) and about 17% of them reported that they were almost absent from school. From the total sample, the mean depression scores and academic performance scores were 10.2 and 3.1, respectively. From the total sample, 9.3% and 28.3% reported that they were additive to electronic games and social media use (Table 1).

The bivariate analyses revealed several significant differences in the prevalence of addiction to playing electronic games and using social media across various sociodemographic, life style, online use behaviors, and school level groups (Table 1). Higher prevalence of addiction to playing electronic games and using social media were identified among male students, students whose mothers were illiterate, and sleep less than normal hours. Reports of addiction to playing electronic games and using social media use were significantly more frequent among students whose daily game play and daily social media use was > 4 h. and students who were almost absent from the school.

Reports of the higher prevalence of addiction to playing electronic games were found to be higher among students who did not follow the recommended physical activities and students who reported that their relationship with teachers was poor. This study found that there were significant variations in the mean depression scores and academic achievement in relation to the addiction to social media and electronic game use (Table 1).

### 3.2 Multivariate Analysis

#### 3.2.1 Association Between Depression and Academic Performance With Addiction to Playing Electronic Games and Using Social Media

Table 2 shows the association between depression and academic performance with addiction to playing electronic games and using social media among school‐going adolescents. For one unit increase in depression scores, the odds of addiction to playing electronic games and using social media increase by a factor of 1.14 and 1.12. Additionally, it was found that for one unit an increase in academic performance scores, the odds of addiction to playing electronic games and using social media decrease by a factor of 0.67 and 0.71.

**Table 2:** Multivariate logistic regression analysis for the associations of depression, and academic performance with addiction to playing electronic games and using social media along with other sociodemographic, lifestyle and online use behavior, and school level related characteristics (*n* = 800).

Characteristics	Addiction to playing electronic games		Addiction to using social media	
	aOR	95% CI	aOR	95% CI
Gender (ref = female)				
Male	2.01^c^	1.84–4.45	1.98^c^	1.37–4.01
Educational level, grade (ref = 10th)				
9th	0.59	0.17–2.29	0.96	0.31–1.82
Age, yrs (ref = 15)				
14	0.62	0.23–1.51	0.79	0.30–1.96
Mothers' education (ref = higher education)				
No education	3.37^b^	2.08–8.20	3.08^b^	1.77–5.45
Primary	1.44	0.67–3.31	1.18	0.71–2.21
Secondary	2.10^b^	1.10–3.81	1.31	0.67–2.35
SES (ref = rich)				
Poor	1.43	0.69–3.11	1.18	0.59–2.23
Middle	1.07	0.46–2.48	1.07	0.43–2.33
Family type (ref = Joint)				
Nuclear	0.61	0.30–1.10	0.43	0.14–1.99
Sleeping duration (ref = less than normal)				
Normal	0.66^c^	0.25–0.78	0.51^c^	0.39–0.86
More than normal	0.90	0.53–1.69	0.90	0.49–2.01
Daily game play, h (ref = < 4)				
< 2	0.02^a^	0.01–0.14	0.11^b^	0.03–0.33
2–4	0.07^a^	0.03–0.19	0.77	0.42–1.63
Daily social media use, h (ref = > 4)				
< 2	0.24^b^	0.10–0.44	0.09^a^	0.04–0.25
2–4	0.60	0.24–1.46	0.24^b^	0.15–0.62
Preferred device (ref = mobile phone)				
Other	0.79	0.49–1.15	0.92	0.59–1.49
Recommended physical activities (ref = yes)				
No	2.44^b^	1.54–3.88	1.91^c^	1.30–3.01
Types of school (ref = private				
Government	1.22	0.72–2.13	1.08	0.51–2.31
School attendance activities (ref = never absent)				
Almost absent	2.11^b^	1.33–3.61	1.67^c^	1.05–2.51
Sometimes	1.22	0.80–1.91	1.13	0.60–2.18
Teacher‐student relationship (ref = good)				
Poor	1.29	0.61–2.83	1.52	0.81–3.29
Neutral	1.32	0.41–3.78	1.41	0.61–3.11
Depression	1.14^a^	1.04–1.31	1.12^b^	1.01–1.19
Academic performance	0.67^a^	0.29–0.75	0.71^b^	0.34–0.79

*Note:* a, b and c indicate *p* < 0.001, *p* < 0.01 and *p* < 0.05.

Abbreviations: aOR = adjusted odds ratio, CI = confidence interval.

#### 3.2.2 Associations Between Other Covariates With Addiction to Playing Electronic Games and Using Social Media

Table 2 further demonstrates the association between additional variables and social media and game addiction in adolescent students. The likelihood of becoming addicted to social media and games was shown to be 1.98 times (95% CI = 1.37, 4.01) and 2.01 times (95% CI = 1.84, 4.45) higher in male students, respectively. Normal sleep patterns and using social media and games for less than 2 h were associated with lower risk of being addicted to social media and electronic games. Additionally, a mother's lack of education and students' almost‐absenteeism from school were associated with an increased likelihood of being addicted to social media and electronic games.

## 4 Discussion

### 4.1 Major Findings

To the best of our knowledge, this is the first study in a setting of a developing country to investigate the association between depressive symptoms, academic achievement, and an addiction to social media and electronic games play among adolescents studying in high school. There were six significant findings. First, a comparatively higher prevalence of electronic games addiction (9.3%) was noted. Second, more than a quarter of social media users have a social media addiction. Third, a higher score of depression was discovered. Fourth, the mean score for academic performance was lower. Fifth, it has been found that an addiction to social media and electronic games was linked to depression and academic performance.

Lastly, when additional sociodemographic, lifestyle, online behavior, and school‐level factors were considered, male children, children whose mothers were uneducated, longer durations of playing electronic games and using social media, sleeping shorter than normal, not adherent to recommended physical activity, and almost absent from school were all associated with a higher risk of being addicted to playing games and using social media.

### 4.2 Comparing the Results With Previous Findings

While no comprehensive study was done among school‐age Bangladeshi adolescents, our results, which showed a higher prevalence of electronic gaming addiction, were in line with other studies that used the same IGD‐SF9 measuring tool and were conducted in several developing countries, like Nepal (18.9%) [34], India (6.5%) [35], Turkey (8.5%) [36] and Malaysia (3.5%) [37]. The finding that Bangladeshi adolescents enrolled in school had higher prevalence of game addiction raised concerns about an important public health issue.

This study's sample data showed that almost one in four adolescents were addicted to social media, which is like the findings of a prior study (20.1%) that used the same threshold of ≥ 18 out of 30 on the BFAS scale among Bangladeshi high school adolescents [32]. Studies show that adolescents in a number of worldwide developing contexts, including Turkey (24.4%) [38], India (23.5%) [39], and Tunesia (13.6%) [40] have a high frequency of social media addiction. The target demographic such as age, gender, sample size, and social and cultural variations may account for the disparities in the prevalence of social media addiction between this study and other ones. Given the higher prevalence of social media addiction raises another concern for public health. The findings imply that it is preferable for the high school's legislative body to implement strict guidelines for encouraging responsible use and lessening the negative consequences of this issue on pupils.

According to the current study, a high score of depression was found among school adolescents. Previous studies carried out using PHQ‐9 in several low resource settings such as in China (13.5%) [41], Nepal (44.2%) [42], India (29.6%) [43], Ethiopia (28.6%) [44], and Kenya (33%) [45] agree with this finding. The identical screening instruments—patient health questionnaires—used in both the prior and current investigations may be the cause of the agreement. Furthermore, the use of the same study populations in the earlier and current studies could be another explanation for their similarities.

Primary education in Bangladesh has advanced significantly, but secondary education has not yet reached the anticipated levels of development [46]. A significant portion of high school students in Bangladesh are unable to pass the Secondary School Certificate (SSC) or equivalent examinations every year [46]. One of the main reasons why so many pupils in this nation failed the secondary school certification exam is the increased frequency of poor academic performance. Accordingly, this survey found that the mean score for academic performance was lower. The results thus point to the necessity of putting in place a suitable counseling plan to monitor the academic progress of the underachievers.

Our study demonstrated a link between adolescent depression and academic performance with electronic gaming addiction. This is consistent with several other research that investigated the potential negative consequences of electronic game addiction on depression and academic performance in adolescents in a variety of international settings [47, 48]. Thus, our results are in line with these findings and extend them to a different sample.

The causal mechanisms for the association between game addiction and depression is that excessive gaming creates an environment where individuals are often isolated in the physical world, such as in their room a lot; they may have withdrawn from friends and family, and often, exercise is sparse. This environment and lifestyle can lead to feelings of depression. Potential mechanisms of the association between game addiction and academic performance encompasses detrimental effects on cognitive processes, disturbances to learning patterns, a reduction in study duration, and deficiencies in focus, social engagement, and interpersonal skills [49]. School and health authorities should take proactive measures to raise awareness and implement educational programs considering the concerning correlation between depression and academic achievement with addiction to playing electronic games among adolescents enrolled in school. In addition, parents must closely monitor their children's use of electronic gaming gadgets at home.

Consistent with several other prior research [50, 51], our study indicated that addiction to social media (Facebook) was associated with higher scores of depressive symptoms in school‐age adolescents. Social media addiction can lead to isolation and loneliness, comparison and jealousy, sleep difficulties, cyberbullying, fear of missing out, and loneliness, all of which can raise the risk of depression [52]. Additionally, this study demonstrated that the academic performance of adolescent pupils was statistically significantly impacted by social media addiction. This result is consistent with several other earlier research from Nigeria [53], Turkey [54], and India [55], which demonstrate a link between poor educational outcomes and social media addiction. Conversely, other earlier research revealed no connection between adolescent students' academic achievement and social media addiction [56, 57]. There could be several reasons for the differences in these results, including the target age group of the adolescents, the qualitative methods used for data collection, the GPA measurement, and the inclusion of social media platforms other than Facebook, like Instagram, YouTube, and WhatsApp, which could obscure the true effects of the current Facebook addiction.

As a result, it is critical that school administrators take proactive measures to support adolescents who are reliant on these networks and educate them through seminars on the dangers of social media addiction. To prevent setbacks in students' academic performance, the researcher suggested that social media be used for educational purposes as well; that social networking sites be expanded, and new pages created to enhance academic activities; and that parents and teachers should keep an eye on their children's use of these sites. This aims to strike a balance between students' use of social media and their academic pursuits to prevent academic setbacks and lessen student depression.

Gender differences in social media and electronic game users are one of the factors that could lead to a rise in social media and gaming addiction. According to our research, male students were noticeably more likely than female students to become addicted to electronic games. Previous studies considering samples of adolescents [58, 59, 60]. have shown the same results. Maladaptive cognitions such as the overvaluation of virtual rewards may explain the higher prevalence of gaming addiction among males [59]. Consistent with earlier research, our findings showed that male students were more addicted to social media use than female students [61, 62]. The duration of sleep and social media and game addiction were found to be significantly associated. This is consistent with earlier research done in different areas of the world that found that sleep durations of less than 6 h per day are linked to addiction to electronic games [11, 63] or social media use [64].

The duration of daily use is a crucial component in determining the impact on social media and game addiction. According to this study, playing electronic games or using social media for longer than 4 h every day raised the chance of addiction. The current study's findings are in line with previous studies [65, 66]. There was a significant association between a mother's educational background and her children's addiction to social media and gaming. According to a previous systematic assessment [67], people who have a mother with little formal education—rather than their father—are more likely to be addicted to social media or gaming.

According to this study, there was a significant association between the recommended level of physical activity and a decline in games and social media addiction. Studies [14, 68, 69] that corroborate our findings show that physical activity has a significant role in addressing the issue of social media or game addiction. Physical activity may be beneficial for people who are addicted to games or social media because it offers a constructive way to release tension and anxiety and encourages self‐control and self‐regulation. Addiction to social media or gaming can have a detrimental effect on students' attendance at school. Research has indicated that increased use of social media and gaming platforms is associated with higher absences from school. Our investigation yielded findings that were consistent with earlier research [70].

### 4.3 Strengths and Limitations

The major strengths of this study are: (1) the first study to examine the relationship between depression and academic achievement with two forms of technology addiction, such as an addiction to social media and electronic games, among Bangladeshi adolescents enrolled in school; (2) it took into account both private and public schools, involves both genders, and uses a reliable assessment tool for both depression and gaming and social media addiction; and (3) pertinent confounders have been selected for this analysis based on their theoretical and empirical significance in a variety of studies.

In addition, the following should be kept in mind while interpreting the results: *First*, as the study was cross‐sectional in nature, it was not possible to establish a causal relationship between the desired outcomes and addiction to electronic games or social media. In the future, longitudinal research will be necessary to prove causation. *Second*, the study's restricted study region (Rajshahi City Corporation) severely restricts the study's ability to generalize to the entire nation and beyond. Future research must use designs with larger and more representative samples to address these limitations. *Third*, certain aspects of gaming—like game genres, gaming budget, play environments, and play schedule—were not evaluated. *Fourth*, screening questionnaires were used to gather data on depression symptoms, social media addiction, and gaming addiction rather than organized clinical interviews, which would have allowed for more accurate diagnostic evaluations. However, questionnaires rather than structured interviews are frequently used in epidemiological surveys due to budgetary constraints.

*Fifth*, the memory of some information was hazy, like academic achievement. *Finally*, the current study solely evaluated Facebook addiction, which may not be representative of all addictions to social media. Facebook is a type of social media that encompasses numerous websites (Twitter, Google+ , Line, etc.). Consequently, more research should be done to investigate alternative social media platforms that may mirror social media addiction.

## 5 Conclusions

In conclusion, our study shows that a substantial number of school‐age adolescents in Bangladesh are addicted to social media and electronic games. The likelihood of depression and academic achievement in adolescents was found to be associated with game addiction and social media addiction. An increased likelihood of developing an addiction to playing games and using social media has been associated to male children, children whose mothers were illiterate, longer periods of playing electronic games and using social media, shorter sleep durations than usual, noncompliance with recommended physical activity, and almost total absence from school. Future interventions could take these aspects into consideration to reduce excessive game playing and social media use. Future longitudinal research will be required to demonstrate the causality of these relationships.

## Author Contributions

**Mosiur Rahman:** conceptualization, data curation, formal analysis, visualization, writing – original draft, writing – review and editing, project administration, supervision, investigation, methodology, software, validation, funding acquisition, resources. **Prosannajid Sarkar:** visualization, writing – review and editing, software. **Syed Emdadul Haque:** investigation, project administration, writing – review and editing. **G. M. Rabiul Islam:** writing – review and editing, investigation. **Md. Nuruzzaman Haque:** investigation, writing – review and editing. **Tapan Kumar Roy:** validation, resources, software, investigation, writing – review and editing. **Farhana Akhter Liza:** investigation, writing – review and editing. **Anika Tabashsum:** investigation, writing – review and editing. **Md. Rashed Alam:** software, validation, resources. **Mahmudul Hasan:** software, validation, resources. **Izzeldin Fadl Adam:** investigation, writing – review and editing, validation, software. **Nguyen Huu Chau Duc:** methodology, project administration, software, and resources. **Chowdhury Mashrur Mahdee:** resources and software. **Jannatul Fardos Asha:** software and resources.

## Conflicts of Interest

The authors declare no conflicts of interest.

## Transparency Statement

The lead author Mosiur Rahman affirms that this manuscript is an honest, accurate, and transparent account of the study being reported; that no important aspects of the study have been omitted; and that any discrepancies from the study as planned (and, if relevant, registered) have been explained.

## Data Availability

The data that support the findings of this study are available from the corresponding author upon reasonable request.
